# Scaling the Need, Benefits, and Risks Associated with COVID-19 Acute and Postacute Care Rehabilitation: A Review

**DOI:** 10.1155/2020/3642143

**Published:** 2020-08-26

**Authors:** Sayed Zulfiqar Ali Shah, Mohammad Nasb, Min Lu, Liangjiang Huang, Yizhao Wang, Hong Chen

**Affiliations:** ^1^Department of Rehabilitation Medicine, Tongji Hospital, Tongji Medical College, Huazhong University of Science and Technology, Wuhan 430030, China; ^2^WHO Collaborating Center for Training and Research in Rehabilitation, Tongji Hospital, Tongji Medical College, Huazhong University of Science and Technology, Wuhan 430030, China

## Abstract

Coronavirus is an RNA virus, which attacks the respiratory system causing complications including severe respiratory distress and pneumonia and many other symptoms. Recently, a novel coronavirus (COVID-19) outbreak emerged in Wuhan, which caused a significant number of infections in China and resulted in a global pandemic. The main aim of this study is to review and summarize the evidence regarding the supportive role of physical rehabilitation techniques in managing COVID-19-associated pneumonia. In this review, we also emphasize the use of rehabilitation techniques in the management of pneumonia in COVID-19-infected patients. Based on the evidence presented, we conclude that certain physical rehabilitation techniques and modalities could be of great support in the management of COVID-19-associated pneumonia. The safety of staff and patients when applying rehabilitation intervention requires attention. The combination of physical rehabilitation and medical treatment would result in improved treatment outcomes, faster recovery, and shorter hospital stay. Many rehabilitation techniques are safe and feasible and can be easily incorporated into the management protocol of COVID-19 victims. Decisions of early rehabilitation induction should be based on the patient's medical condition and tolerability.

## 1. Background

Coronaviruses are crown-like enveloped viruses which belong to the Coronaviridae family [1, 2]. So far, six coronaviruses (229E, OC43, MERS-CoV, SARS-CoV, HKU1, and NL63) have been successfully identified [3] and can cause deadly infections in humans. The coronavirus severe acute respiratory syndrome (SARS) outbreak was first identified in 2002-2003 in Guangdong Province of China, which infected 8477 people in many Asian and European countries, as well as the United States. The disease was quickly transmitted from person to person via contact in households and healthcare settings, and later, the outbreak spiked massively in communities [4]. In 2012, MERS-CoV had caused an outbreak in the Middle East and was therefore named Middle East Respiratory Syndrome (MERS) [5].

Coronaviruses are widely distributed around the globe. The CoV genetic variations, frequent genetic recombination events, multiple animal hosts, and animal-to-human interface are increasing the chances of the emergence of novel and more virulent coronaviruses [6]. A novel and more virulent coronavirus termed SARS-CoV-2 was identified in Wuhan, Central China, in January 2020 when an outbreak of pneumonia of an unknown aetiology emerged [7]. The outbreak of this novel coronavirus disease, COVID-19, has resulted in a global pandemic, and so far, 3,939,119 cases have been reported worldwide, with 274,917 global deaths (5/10/2020) [8].

The purpose of this study was to review the evidence regarding the supportive role of treatment options available in physical rehabilitation to manage COVID-19 pneumonia effectively. We are also aiming to present our rehabilitation experience from China, the potential of local COVID-19 transmission, staff safety, and infection control during rehabilitation interventions. Evidence strongly supports that many rehabilitation techniques including chest physiotherapy and physical therapy modalities can be of great support to manage COVID-19-associated pneumonia [9, 10]. Combined medical and rehabilitation therapy would result in improved treatment outcomes in coronavirus-infected patients.

## 2. Methods

Potential relevant articles were identified through a PubMed, Ovid, and Google Scholar search. English and Chinese language studies of mixed designs were reviewed, focusing on different aspects of rehabilitation of coronavirus-infected patients.

## 3. Diagnosis

**Figure 1:**
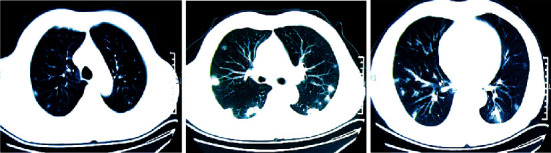
Chest CT scans of a COVID-19 pneumonia patient on day 5 of the onset of symptoms.

**Figure 2:**
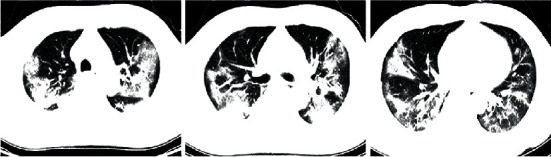
Chest CT scans of a COVID-19 pneumonia patient on day 9 of the onset of symptoms.

**Figure 3:**
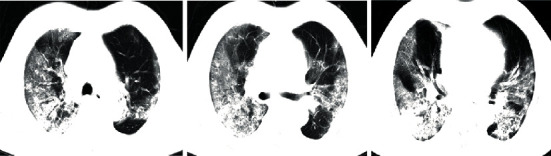
Chest CT scans of a COVID-19 pneumonia patient on day 14 of the onset of symptoms.

SARS-CoV-2 can be isolated through RT-PCR and generation sequencing of the nucleic acid extracted from the samples collected from the respiratory system of the infected patients. Investigations for the diagnosis should include routine complete blood count, serum analysis, and computed tomographic (CT) scans for assessing pulmonary changes. Clinical signs and symptoms, chest radiography, and ruling out of other pathogens play an important role in the diagnosis of coronavirus-associated pneumonia [11, 12]. In Figures 1–3, we have shown CT images of three cases of pneumonia taken at the onset of symptoms featuring different stages of the disease.

## 4. Coronavirus Mechanism of Transmission

It is clear that SARS-CoV-2 is more transmissible than SARS-CoV, but transmission dynamics, mode, and route of transmission remain unclear [13]. Along with the zoonotic transmission to humans due to sustained human-to-animal contact, human-to-human spread of coronaviruses (SARS and MERS-CoVs) was also confirmed. Human-to-human transmission was mostly seen when there was sustained contact with infected individuals, for example, in healthcare settings and households [14]. It is also suggested that for human coronavirus infection, an intermediate host may not be required [15]. Coronavirus transmission through respiratory droplets, human contact, or contaminated objects like clothes and utensils was confirmed by many studies [16, 17].

### 4.1. COVID-2019 Nosocomial Transmission

COVID-19 has local transmission, and healthcare workers can transmit the virus to patients and wards if proper measures during treatment are not taken. Earlier, it was reported that in a cohort of 138 pneumonia patients, 17 patients and 40 healthcare providers were thought to have been infected within the hospital [12]. Chest physiotherapy and faecal management have been declared as a high-risk procedure [18]. The exhaled or leaked air during noninvasive ventilation (NIV) can propagate virus dispersion, thus increasing the facility-based infection rate [19]. Simonds et al. addressed the droplet dispersion during CPT, NIV, oxygen therapy (O^2^), and nebulizer use in patients with lung disease and coryzal symptoms. It was reported that CPT and NIV are droplet-generating techniques but not aerosol-generating techniques and produced droplets of >10 *μ*m in size. A nebulizer was declared as aerosol-generating therapy. The study recommended that healthcare staff performing NIV and CPT within 1 m of the patients should use high protective measures [19].

### 4.2. Healthcare Staff Safety and Infection Control

All staff must be trained in infection control measures, proper donning and doffing of personal protective equipment (PPE), correct hand washing procedure, and wearing head and shoe covers, gloves, plastic aprons or gowns, and certified N95 masks with 95% or more filtering ability of aerosol particles of 0.3 *μ* [18]. Previous experience from SARS has shown that rehabilitation specialists should be cautious because of potential aerial transmission while applying NIV therapies [18]. A sealed plastic bag should be used to cover the mouth during expectoration to prevent the spread of infection [20]. Since the virus spreads through respiratory droplets or aerosol particles, for staff safety, a positive air-purifying respirator (PAPR) is highly recommended. PAPR is a battery-operated device, which expels filtered air and helps in the prevention of inhaling the virus, which is necessary while performing suctioning or other techniques generating such particles and droplets. Heat generation, movement restrictions, device weight, and non-air-conditioned environments make wearing PAPR difficult for staff [21]. Care should be taken while performing CPT and postural drainage as these techniques produce coughing and assist in expectorating secretions. Therefore, the above techniques and use of nebulizers should be carried out in closed rooms, with staff wearing PPE and PAPR. Faecal management and the disposal of diapers should be done while wearing PPE [22]. The N95 mask increases the breathing rate, inducing early fatigue and even dizziness in some staff members. The PPE use was especially troublesome in pregnant staff and in tropical regions [18]. Healthcare workers with respiratory problems who found wearing PPE such as N95 difficult, or staff suffering from wet eczema who found that it could become worse by following some of the protective measures including frequent hand washing and gloves, should not be compelled to work in areas requiring high PPE protective measures [23]. Research has shown that the virus can transmit locally and through surfaces of physiotherapy equipment including modality probes, pads, therapy balls, and hot packs. It is recommended to clean surfaces with 62-71% ethanol which inactivates the virus within a minute; otherwise, it could remain on the surface for a longer period of time [24].

## 5. Rehabilitation

### 5.1. Rehabilitation Necessity

Longitudinal studies have reported that due to long periods of bed rest, some pathological changes including pulmonary fibrosis, alveolitis, atelectasis, muscular weakness, and other neuromuscular problems may persist [25]. The aforementioned problems in SARS patients impair the quality of life, cause dyspnea on exertion, and lead to residual pulmonary dysfunction [26, 27]. We speculate that extensive lung damage, multiple organ failure in some COVID-19 patients, and longer bed rest may cause residual pathological problems, physical dysfunction, and functional limitations which provide strong evidence for the incorporation of rehabilitation into COVID-19 management.

### 5.2. Rehabilitation Evidence

Common problems identified in COVID-19 patients that could be managed by rehabilitation specialists in the postacute phase include musculoskeletal pain, joint pain, reduced range of motion, muscular weakness, neuropathy and myopathy, pulmonary dysfunction, dysphagia, dyspnea, confusion, and impaired activities of daily living. It remains unclear whether the impact of the problems mentioned would persist in the chronic phase [28]. Long-term exposure to corticosteroid therapy can cause many musculoskeletal problems. After the SARS outbreak in China at the end of 2002, Chinese and international literature reported many cases of avascular osteonecrosis (AVN) [29–31]. Drug therapy represents an essential treatment of pneumonia, whereas some other treatments are mainly supportive, including but not limited to intravenous hydration, supplementary oxygen, and chest physiotherapy [32]. Chest physiotherapy is commonly used as an adjuvant treatment for pneumonia [33]. Chest physiotherapy aids in the treatment of symptoms of several respiratory disorders, which are the most familiar cause of both morbidity and mortality in ICU patients [34]. In such cases, physiotherapy is aimed at improving the patient's respiratory system in addition to accelerating recovery by enhancing airway clearance and decreasing airway resistance in lung conditions accompanied by hypersecretion and increased airway resistance. Chest physiotherapy is a key treatment for individuals with copious secretions (>30 ml per day) and decreased ability to cough [35].

A six-week pulmonary rehab program of discharged 133 SARS patients included aerobic exercise and upper and lower extremity exercise. The experimental group was given pulmonary rehabilitation including aerobic exercise, upper and lower extremity exercises, while the control group received only conventional care, the study reported significant improvement in oxygen consumption, 6-minute walk test and muscle strength in experimental group [36].

### 5.3. Rehabilitation Recommendations, Guidelines, and Experience from China

Chinese experts and frontline COVID-19 doctors developed pulmonary rehabilitation guidelines with short-term goals of relieving dyspnea and anxiety and long-term goals of functional improvement, enhancing quality of life, and bringing the patient to a normal societal level. The Chinese rehabilitation protocol broadly included aerobic exercises and strength, balance, respiratory, and healthcare training, using traditional Chinese medicine. According to Chinese experts, the consensus rehabilitation program should be stopped if the patient has peripheral capillary oxygen saturation (SpO_2_) < 88% or reports chest tightness, palpitations, sweating, and dyspnea, which are considered inappropriate for rehabilitation [37].

Group therapies should be discontinued, and practice should be one-on-one mostly at the bedside [21].

According to consensus-based acute care physiotherapy recommendations developed in Qatar, rehabilitation patients could be categorized into the following six categories: Category A (paralyzed, ventilated, and sedated), Category B (ventilated and minimally sedated), Category C (no mechanical ventilation), Category C.1 (bedridden but minimally conscious), Category C.2 (conscious and active but dependent), and Category C.3 (independent, active, and conscious). Category-wise, rehabilitation interventions were recommended as follows: A: passive range of motion (PROM), positioning; B: positioning, PROM, and graded mobility; and C: C.1: positioning, PROM, and mobilization; C.2: ROM, progressive strengthening, and coordination exercises; C.3: ROM, breathing exercises, and graded ambulation [38].

### 5.4. Early Rehabilitation?

The weaning phase and referral to rehabilitation should be staged and gradual: a gap of three weeks each is suggested for moving ICU patients to an acute care ward and finally rehabilitation. COVID-19 pneumonia patients have to remain immobilized and stay in prone positions for longer hours, due to which early rehabilitation induction may not be tolerable. A sudden drop in saturation, fatigue, and worsening of respiratory symptoms could occur [28].

### 5.5. COVID-19 Acute Care Rehabilitation

#### 5.5.1. Active Cycle of Breathing Technique (ACBT)

**Figure 4:**
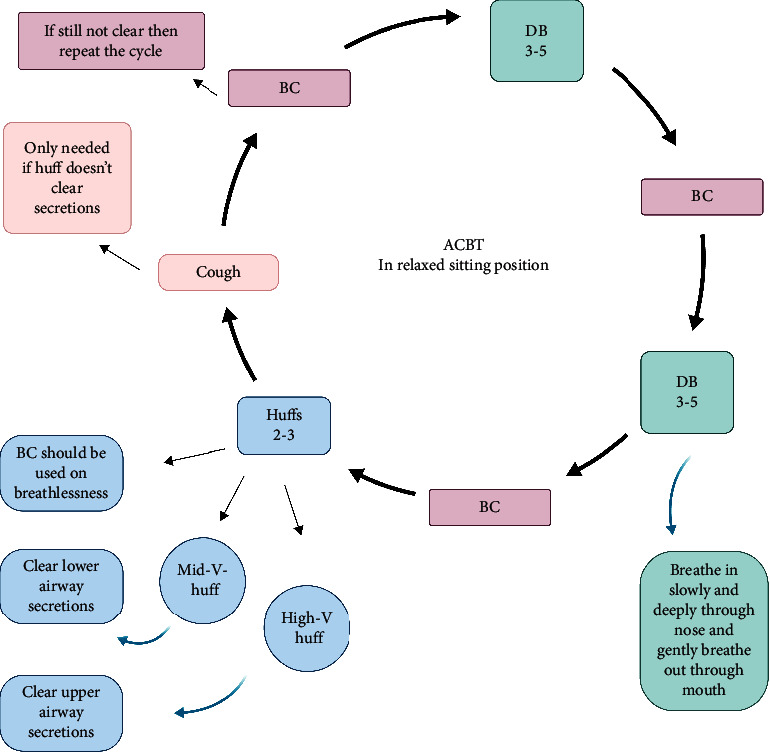
Active Cycle of Breathing Technique (ACBT).

It refers to the technique, which is used to mobilize and clear pulmonary secretions in addition to improving lung function. It includes two phases: the first phase is aimed at relaxing the airways by thoracic expansion exercises and the second phase is the Forced Expiratory Technique (FET), which involves a huff and cough breathing exercise, which is used to help clear secretions from the airways (Figure 4) [39]. Note: as these techniques create droplets, the benefits and risks should be weighed up before applying them [40].

#### 5.5.2. Postural Drainage with Percussion (PD & P)

It is the usage of force of gravity combined with percussion (clapping on the chest and/or back) to release the thick, sticky mucus from the lung peripheries to remove it easily by coughing. Lots of studies have shown its effectiveness in treating pneumonia cases [41, 42]. PD includes comfortably lying in the proper gravity-assisted drainage position depending on the affected lung zones. In each position, the chest wall should be percussed for three to five minutes, followed by deep breathing exercises accompanied by vibrations on expiration and then two to three huffs. The patient should then be encouraged to cough and expectorate the dislodged secretions. This is followed by a short rest interval of controlled breathing between two cycles. This cycle is repeated for each position, respectively. This treatment is usually done twice daily or according to the patient's situation. Each cycle takes approximately 30 minutes [43].

Note: scale the expected improvement first; some positions are difficult to achieve, and also, changing position is associated with droplet generation.

#### 5.5.3. Noninvasive Ventilation (NIV)

NIV therapy through CPAP and HFNO can manage hypoxia, improve respiratory failure, and help in delaying or avoiding endotracheal intubation [44]. Invasive ventilation techniques should be adopted if the patient did not respond to NIV quickly [45]. The application of NIV in a properly ventilated ward with negative pressure could be helpful [46].

#### 5.5.4. Positive Expiratory Pressure (PEP)

It is a device used to offer positive expiratory pressure during expiration ranging from 10 to 25 cmH_2_O. PEP can help in keeping the airways open during expiration which could enable airway clearance.

#### 5.5.5. Continuous Positive Airway Pressure (CPAP)

It is a treatment technique that is aimed at improving the oxygen saturation of patients using a high-pressure gas to provide a constant pressure in the airways in both inspiration and expiration in patients breathing spontaneously. CPAP masks could be placed over the nose or mouth [47]. Brambilla et al. revealed that CPAP could decrease the risk of meeting endotracheal intubation criteria in patients with severe hypoxemic acute respiratory failure caused by pneumonia [48]. Recommendation: it is recommended that if an hour treatment with CPAP/NIV did not bring significant improvement in symptoms, patients should proceed to invasive ventilation [44]. A single-tube CPAP with an exhalation port, antiviral filter, and nonvented mask is highly recommended [45].

#### 5.5.6. Bilevel Positive Airway Pressure (BI-PAP)

It delivers two different levels of pressure throughout the respiratory cycle: a higher pressure with inspiration which is called inspiratory positive airway pressure (IPAP) and a lower one during the expiration which is called expiratory positive airway pressure (EPAP). When the patient begins to inhale or exhale, the device automatically shifts between EPAP and IPAP settings. BI-PAP could enhance comfort for patients who suffer from breathing out difficulty [49].

#### 5.5.7. Suctioning

It is the removal of secretions through negative pressure. The manual techniques mentioned so far are effective to cough up the sputum or mobilize it to the central airways, and from there, it can be easily removed by suctioning with a pressure of 11–16 kPa, or 20 if the secretions are thick, for 15 seconds. The catheter used for suctioning should be smaller than half of the diameter of the endotracheal tube or tracheostomy. The injection of sodium chloride before commencing suction can loosen the secretions in the presence of thick secretions [50]. Recommendation: open circuit suctioning is not recommended as it causes dispersion of respiratory droplets. Disconnection from the ventilator may cause a loss of positive end expiratory pressure and worsen the atelectasis [40].

#### 5.5.8. Changing Posture

A prone position is suggested for 12-16 hours daily. When the PaO_2_/FiO_2_ ratio (P/F) reaches ≥150 mmHg with PEEP ≤ 10 cmH_2_O and FiO_2_ ≤ 0.60 for at least four hours after supine positioning, prone positioning should be stopped [51]. Note: changing position may arouse cough and produce respiratory droplets, so unnecessarily changing posture is not recommended.

#### 5.5.9. Mobility

Passive ROM exercises are recommended to delay muscular weakness, cure reduced ROM due to immobility, and prevent integumentary problems [45]. Note: active mobility is not recommended in acute care, due to the chances of sudden desaturation and respiratory droplet generation.

### 5.6. Chest Physiotherapy Precautions and Contraindications

Postural drainage (PD) is relatively contraindicated in patients with raised intracranial pressure (ICP), fresh spinal injury, recent eye surgery, hemodynamic instability, hernia of the diaphragm, and patients who went through esophageal anastomosis recently. Additionally, precautions should be taken while performing PD in pleural effusion, hemoptysis, pleural edema, and ascites patients. Chest percussion and vibration are relatively contradicted in hemoptysis, neglected tension pneumothorax, pulmonary embolism, open wounds, low platelet count, hemodynamic instability, and fresh thoracic skin graft patients. While performing percussion and vibration alone or together with PD, precaution is required in patients with osteoporosis, rib fractures, and bronchospasm, as well as in recent pacemaker transplant patients [52]. Positive pressure therapies need tremendous caution in unstable hemodynamic conditions and patients with cranial, facial, or oral surgeries [50]. CPAP is contraindicated in severe facial injuries, skull fractures, undrained pneumothorax, paralytic ileus, and surgical emphysema [53]. There is a variety of contraindications to endotracheal suctioning differing from case to case, but the most common are bronchospasm and raised ICP. In cases reporting bleeding disorders, epiglottitis, skull or facial injuries, and laryngospasm, suctioning is contraindicated [54].

### 5.7. Ultrashortwave Therapy (USWT)

Chinese literature has prudently proven that USWT has anti-inflammatory and analgesic effects in pulmonary infection. The application of Curapulse 970™ shortwave diathermy machines and two Radarmed 950+™ microwave machines was performed, and the results were promising for COVID-19 treatment. The treatment session involved the application of the USWT electrode on the anterior and posterior parts of the trunk for 15 m/d till patient discharge [55]. A review of the biological effects of shortwave concluded that based SW has fewer side effects and more benefits, but they also require further research on the side effects [56]. Chinese USWT literature in pulmonary conditions is prevailing because of the wide use of USWT in such conditions. Chinese studies had shown significant results on the use of USWT for different pulmonary conditions in humans and animals. Some of the studies are Zhang et al. [57] on therapeutic effects of USWT on SARS, Zhang et al. [58] on USWT in type 1I respiratory failure, Zhou et al. [59] on USWT in interstitial fibrosis in preclinical models, and Hou et al. who utilized SWD in bactericidal and virucidal problems [60]. Some of these studies claimed to be randomized controlled trials (RCTs), but they were lacking the features of an RCT and also did not provide important details regarding USWT therapy. It has previously been shown that raising the temperature decreases the activity and viability of viruses [61]. Thus, and depending on earlier studies, the utilization of shortwave diathermy could aid in such infectious conditions [60, 62]. Shortwave diathermy (SWD) is among the first physiotherapy modalities, and it remains in use in rehabilitation departments. It has been also used to treat a wide range of conditions [63]. Moreover, pulsed SWD was the third most commonly used physiotherapy in the UK [64]. The therapeutic effects of SWD on the body parts include the production of efficient deep heating in human body structures within the physiological limits [65]. If applied to a specific muscle, SWD roughly raises its temperature to approximately 15.14°C, while the application of SWD on lungs would not cause it to rise to that temperature, as the lungs own an efficient water cooling system in addition to the pulmonary circulation which prevents any substantial rising of local heating. This feature supports the safety of SWD and prevents tissue damage [65]. Moreover, the practical application of SWD showed promising effects on relieving thoracic pain, dyspnea, and cyanosis.

#### 5.7.1. SWD Contraindications

The contraindications for shortwave diathermy include the presence of internal metallic objects in the treatment field, such as an intrauterine device, pacemakers, and metal implants. Moreover, SWD is contraindicated to be applied on the following body parts and conditions: malignancy, pregnancy, menstruation, the eyes, gonads and growing epiphyses, ischemic tissues, hemorrhagic areas, venous thrombosis/phlebitis, cardiac conditions, blood pressure abnormalities, impaired sensation, wet dressings or adhesive tape, and open wounds. In addition, use on a pregnant uterus should be avoided. Due to the risk of tissue burns and necrosis, precautions need to be taken before proceeding with treatment because sweating could cause tissue burn [66]. Moreover, patients should not move during the treatment, as it could result in burning the subcutaneous fat necrosis [67, 68].

Although Chinese literature and some other studies have proven the effectiveness of USWT in pulmonary infection and reported fewer or no side effects, USWT is still debated around the globe. To address this ambiguous situation about the therapeutic efficacy of USWT, we conducted a rigorously designed RCT in Tongji Hospital, Wuhan. The results of the study are promising regarding different aspects of COVID-19 treatment. The study will be published soon.

### 5.8. COVID-19 Postacute Care Rehabilitation

The postacute care phase has been considered as more appropriate for commencing rehabilitation in COVID-19 patients as most of the patients appear to be medically stable at this stage and can tolerate individual rehabilitation programs. ROM, strengthening, balance and coordination exercises, mild treadmill use, and respiratory therapy if needed have been recommended [36, 37]. Moreover, telerehabilitation can be introduced in this phase, offering comparative effectiveness and more safety [37].

### 5.9. Telerehabilitation: A Better Option

There is a lack of evidence to support the use of telerehabilitation during a pandemic. However, telerehabilitation offers online assessment and treatment sessions without exposure of the practitioner to an infection-prone environment. On the other hand, telerehabilitation is a better choice for patients at higher risk of COVID-19 mortality, such as immunocompromised individuals and diabetes patients. E-physio, online assessment, and therapy sessions on webcams could be helpful [23]. With advancement in technology and portable devices, intelligent practice and digital rehabilitation offer more safety and comparable effectiveness is possible today. Downloadable applications are available with video and textual trainings for respiratory exercises. Such devices are effective in offering assessment, feedback, therapy reminders, and treatment progression [37]. Another study also recommended the use of telerehabilitation, training videos, and brochures in order to save PPEs and reduce the spread of infection [20].

### 5.10. Procedures Not to Be Applied in the Acute Phase

COVID-19 patients have reduced respiratory efficiency and less strength of respiratory muscles, so techniques that exhaust such patients should not be applied. The following respiratory therapy procedures are not recommended in the COVID-19 acute phase: pursed lip and diaphragmatic breathing, reexpansion techniques for lungs, use of incentive spirometer, and rib mobilization [45].

## 6. Conclusion

Based on the evidence presented, we conclude that certain physical rehabilitation techniques and modalities could be of great support in managing COVID-19-associated pneumonia. A combination of physical rehabilitation and medical treatment would result in better treatment outcomes, quick recovery, and shorter hospital stays. Early rehabilitation is not recommended because of less tolerability; however if the patient's condition permits, and the benefits outweigh the risks, rehabilitation should be commenced early. Telerehabilitation should be considered in COVID-19 patients, which could offer less staff and patient exposure and more safety, as well as save PPE.
